# Supplementary material for: The Efficacy and Safety Herbal Medicine for Symptom Management After HIFU Treatment in Adenomyosis: A Systematic Review and Meta-Analysis
Source: Pharmaceuticals (Basel). 2025 Jun 4;18(6):843. doi: 10.3390/ph18060843 (PMC12195669; doi:10.3390/ph18060843)
Supplement: Supplementary file 1 [file pharmaceuticals-18-00843-s001.zip › Supplementary File S2. Search strategy used in each database.pdf]

Supplementary File 2. Search strategy used in each database.

Medline via PubMed

	Searches	Results
#1	adenomyosis[Title/Abstract]	4,253
#2	"herbal medic*"[MeSH Terms] OR "medicine, chinese traditional"[MeSH Terms] OR "medicine, kampo"[MeSH Terms] OR "medicine, korean traditional"[MeSH Terms] OR "Korean medicine"[Title/Abstract] OR "Chinese medicine"[Title/Abstract] OR "Oriental medicine"[Title/Abstract] OR "Kampo medicine"[Title/Abstract] OR "herbal medicine"[Title/Abstract] OR "traditional medicine"[Title/Abstract]	95,183
#3	#1 AND #2	**24**

EMBASE

	Searches	Results
#1	'adenomyosis'/exp OR adenomyosis:ti,ab	9,105
#2	'herbal medic*' OR 'medicine, chinese traditional'/exp OR 'medicine, kampo'/exp OR 'medicine, korean traditional'/exp OR 'korean medicine'/exp OR 'chinese medicine'/exp OR 'oriental medicine'/exp OR 'kampo medicinel' OR 'herbal medicine'/exp OR 'traditional medicine'/exp OR 'herbal medic*':ab,ti OR 'medicine, chinese traditional':ab,ti OR 'medicine, kampo':ab,ti OR 'medicine, korean traditional':ab,ti OR 'korean medicine':ab,ti OR 'chinese medicine':ab,ti OR 'oriental medicine':ab,ti OR 'kampo medicine':ab,ti OR 'herbal medicine':ab,ti OR 'traditional medicine':ab,ti	216,573
#3	#1 AND #2	**90**

CENTRAL

	Searches	Results
#1	MeSH descriptor: [adenomyosis] explode all trees	74
#2	MeSH descriptor: [Medicine, Chinese Traditional] explode all trees	1,791
#3	MeSH descriptor: [Medicine, Kampo] explode all trees	64
#4	MeSH descriptor: [Medicine, Korean Traditional] explode all trees	43
#5	#1 AND (#2 OR #3 OR #4)	**0**

CNKI

#1	(SU='adenomyosis'+'子宫腺肌病'+'子宫腺肌症'+'子宫腺肌瘤'+'子宫内膜异位症') AND (SU='Chinese medicine'+'traditional herbal medicine'+'herbal medicine'+'Japanese medicine' +'Traditional medicine'+'Oriental medicine'+'Kampo'+'Korean medicine'+'Complementary medicine'+'herb'+'中药'+'中医药'+'中医'+'灌肠'+'敷贴')	2,587

Wan Fang Database

#1	主题:("adenomyosis" or "子宫腺肌病" or “子宫腺肌瘤” or “子宫腺肌症”) and 主题:("高强度聚集超声" or "HIFU" or "high intensity focused ultrasound" or "focused ultrasound ablation surgery") and 主题:("Chinese medicine" or "traditional herbal medicine" or "herbal medicine" or "Japanese medicine" or "Traditional medicine" or "Oriental medicine" or "Kampo" or "Korean medicine" or "Complementary medicine" or "herb" or "中药" or "中医药" or "中医" or "灌肠" or "敷贴")	36

Chinese Scientific Journal Database (VIP)

#1	M=("adenomyosis" or "子宫腺肌病" or “子宫腺肌瘤” or “子宫腺肌症”) AND M=("高强度聚集超声" or "HIFU" or "high intensity focused ultrasound" or "focused ultrasound ablation surgery") AND M=("Chinese medicine" or "traditional herbal medicine" or "herbal medicine" or "Japanese medicine" or "Traditional medicine" or "Oriental medicine" or "Kampo" or "Korean medicine" or "Complementary medicine" or "herb" or "中药" or "中医药" or "中医" or "灌肠" or "敷贴")	9

Oriental Medicine Advanced Searching Integrated System (OASIS)

#1	자궁선근증	8

Korean studies Information Service System (KISS)

#1	자궁선근증 AND 한약	5

Korea Citation Index (KCI)

#1	자궁선근증 AND 한약	4

Research Information Sharing Service (RISS)

#1	자궁선근증 AND 한약	0

Korean Medical database (KMbase)

#1	자궁선근증 AND 한약	2
