# Supplementary material for: The Efficacy and Safety Herbal Medicine for Symptom Management After HIFU Treatment in Adenomyosis: A Systematic Review and Meta-Analysis
Source: Pharmaceuticals (Basel). 2025 Jun 4;18(6):843. doi: 10.3390/ph18060843 (PMC12195669; doi:10.3390/ph18060843)
Supplement: Supplementary file 1 [file pharmaceuticals-18-00843-s001.zip › Supplementary File S3. Frequency of herb(250604).pdf]

Supplementary File 3. Frequency of herb.

Frequency	Herb
9	Angelica Gigas Root (Angelicae gigantis radix)
6	Sparganium Rhizome (Sparganii rhizoma), Zedoary (Curcumae rhizoma), Red Peony Root (Paeoniae radix), Cnidium Rhizome (Cnidii rhizoma), Corydalis Tuber (Corydalis tuber)
5	Poria (Poria sclerotium), Myrrh (Myrrha), Cinnamon Twig (Cinnamomi ramulus), White Peony Root (Paeoniae radix alba)
