# Supplementary material for: The Efficacy and Safety Herbal Medicine for Symptom Management After HIFU Treatment in Adenomyosis: A Systematic Review and Meta-Analysis
Source: Pharmaceuticals (Basel). 2025 Jun 4;18(6):843. doi: 10.3390/ph18060843 (PMC12195669; doi:10.3390/ph18060843)
Supplement: Supplementary file 1 [file pharmaceuticals-18-00843-s001.zip › Supplementary File S4. Detailed information regarding the HIFU procedures .pdf]

Supplementary File 4. Detailed information regarding the HIFU procedures

First author (year)	Intervention
An(2022)[15]	(1)HIFU (Chongqing HIFU Medical Technology Co., Ltd., JC200A. 100~400W, 0.94MHz, Focal length 150mm, Focal temperature 60~100°C, Scanning speed 3mm/s, Layer spacing 5mm)<br>(2)Anti-inflammatory drugs<br>(3)Anti-infective drugs
Cai(2020) [16]	(1)HIFU (Chongqing HIFU Medical Technology Co., Ltd., JC200)
Dong(2024) [17]	(1)HIFU (Chongqing HIFU Medical Technology Co., Ltd., JC200. 0~400W, 0.96MHz, Diameter 200 mm, Focal length 140 mm)<br>(2)Antibiotic drugs
Pang(2022) [18]	(1)MRgHIFU (GE 750W 3.0T, ExAblate2100, InSightec, Israel, 0.9~1.1MHz, 100~160W, Diameter 2cm, Layer spacing 5mm))
Peng(2021) [19]	(1)HIFU (90~400W, 0.9MHz, Focal length 330mm)<br>(2)LNG-IUS
Shi(2023) [20]	(1)HIFU (Chongqing HIFU Medical Technology Co., Ltd., JC200. 350~400W)
Wang(2025) [21]	(1)HIFU (Chongqing HIFU Medical Technology Co., Ltd., JC200. 350~400W)<br>(2)Sedative and analgesic drugs (Midazolam and Fentanyl)<br>(3)Oral or intramuscular analgesics (If the postoperative pain VAS >7 points)
Xu(2019) [22]	(1)HIFU (Shenzhen ProHuiRen Medical Co., Ltd, PRO2008. 280~300W, Depth 166mm, Layer spacing 2mm)
Xue(2023) [23]	(1)HIFU (Chongqing HIFU Medical Technology Co., Ltd., JC200. 0~400W, 6000~9000W/cm^2, 0.8 MHz, Focal length 145mm, Layer spacing 2~11mm)<br>(2)Antibiotic drugs (3~7 d after HIFU)
Yi(2024) [24]	(1)HIFU (Chongqing HIFU Medical Technology Co., Ltd., JC200D. 0.96MHz, Diameter 200mm, Focal length 155mm)<br>(2)GnRH-a (Leuprorelin acetate microspheres, Shanghai Livzon Pharmaceutical Co., Ltd., 3.75 mg × 1 bottle/box, national medicine standard H20093852. for 3 months, subcutaneously injected)
Yu(2017) [25]	(1)HIFU (Shenzhen ProHuiRen Medical Co., Ltd, PRO2008. 200~300W, Focal time 0.15~0.20s, Interval time 0.1s, Number of strikes 12~15 times)<br>(2)Analgesics, ibuprofen sustained-release capsules (Sino-US Tianjin SmithKline Pharmaceutical Co., Ltd., national medicine standard H10900089)<br>(3)Indomethacin suppositories (Hubei Dongxin Pharmaceutical Co., Ltd., national medicine standard H42021462)
Zhang(2021) [26]	(1)HIFU<br>(2)GnRHa (3.75mg. Subcutaneously injected)
Zhang(2023) [27]	(1)HIFU (Chongqing HIFU Medical Technology Co., Ltd., JC200. 0.96MHz, Diameter 200mm, Focal length 140mm)
Zhou(2021) [28]	(1)HIFU (280~300W, It was performed three times, with a one-week interval between treatments)

VAS = Visual Analogue Score, d = day, m = month, T = tablet, HIFU = High intensity focused ultrasound, MRgFUS = MRI scanner and high-intensity focused ultrasound knife treatment system, LNG-IUS = Levonorgestrel-Releasing Intrauterine System, GnRHa = Gonadotropin-Releasing Hormone agonist
