# Supplementary material for: The Efficacy and Safety Herbal Medicine for Symptom Management After HIFU Treatment in Adenomyosis: A Systematic Review and Meta-Analysis
Source: Pharmaceuticals (Basel). 2025 Jun 4;18(6):843. doi: 10.3390/ph18060843 (PMC12195669; doi:10.3390/ph18060843)
Supplement: Supplementary file 1 [file pharmaceuticals-18-00843-s001.zip › Supplementary File S5. Adverse events.pdf]

Supplementary File 5. Adverse events

First author (year)	Group	N (Total)	N (Adverse events)	Details
An(2022)[15]	E	58	4	Skin toxicity (n=2), Nerve damage (n=1), Periosteal injury (n=1)
	C	58	7	Skin toxicity(n=3), Nerve damage (n=1), Gastrointestinal reactions (n=2), Periosteal injury (n=1)
Pang(2022) [18]	E	30	3	Abdominal pain (n=1), Skin burn(n=1), Vaginal bleeding (n=1)
	C	30	5	Abdominal pain (n=2), Skin burn (n=1), Vaginal bleeding (n=2)
Peng(2021) [19]	E	62	7	NR
	C	62	12	NR
Wang(2025) [21]	E	38	15	NR
	C	39	31	NR
Xu(2019) [22]	E	50	NR	Lower abdominal pain : (2.0 ± 0.94)d, 0.5~5d
	C	70	NR	Lower abdominal pain : (4.22 ± 1.70)d, 0.5~10d
Xue(2023) [23]	E	55	10	Vaginal discharge (n=1), Skin burn (n=3), Pain (n=6)
	C	55	14	Vaginal discharge (n=2), Skin burn (n=4), Pain (n=8)
Yu(2017) [25]	E	30	2	Vaginal bleeding (n=2)
	C	30	1	Vaginal bleeding (n=1)
Zhang(2023) [27]	E	55	5	Abdominal pain (n=2), Vaginal bleeding (n=1), Nausea and vomiting (n=2)
	C	55	7	Fever (n=1), Abdominal pain (n=2), Vaginal bleeding (n=2), Nausea and vomiting (n=2)

E = Experimental group, C = Control group, NR = Not Recorded
