# Supplementary material for: The Efficacy and Safety Herbal Medicine for Symptom Management After HIFU Treatment in Adenomyosis: A Systematic Review and Meta-Analysis
Source: Pharmaceuticals (Basel). 2025 Jun 4;18(6):843. doi: 10.3390/ph18060843 (PMC12195669; doi:10.3390/ph18060843)
Supplement: Supplementary file 1 [file pharmaceuticals-18-00843-s001.zip › Supplementary File S6. Outcome measurement & Result (p -value).pdf]

Supplementary File 6. Outcome measurement & Result(p -value)

First author (year)	Outcome measurement & Result(p -value)
An(2022)[15]	(1)Dysmenorrhea score(VAS): E<C [a](f/u 6m)<br>(2)Blood test indicator<br>a) CA125: E<C [a](f/u 3m, 6m)<br>(3)Endometrial lesion volume: E<C [a](f/u 6m)<br>(4)Menstrual volume: E<C [a](f/u 6m)<br>(5)Adverse events: E<C [b]
Cai(2020) [16]	(1)Dysmenorrhea score: E<C [a]<br>(2)Blood test indicator(f/u 3rd d after the end of the 1st menstrual cycle)<br>a) FSH: E<C [a]<br>b) E2: E<C [a]<br>c) TNFα: E<C [a]<br>d) CRP: E<C [a]<br>e) SAA: E<C [a]<br>(3)TCM syndrome score: E<C [a]<br>(4)Menstrual volume: E<C [a]
Dong(2024) [17]	(1)Dysmenorrhea score<br>a) VAS: E<C [a]<br>b) CMSS: E<C [a]<br>(2)TER: E>C [a]<br>(3)Uterine volume: E<C [a](f/u 14th d of menstruation)<br>(4)Endometrial thickness: E<C [a](f/u 14th d of menstruation)<br>(5)Chronic pelvic pain score: E<C [a]
Pang(2022) [18]	(1)Dysmenorrhea score(VAS): E<C [a](f/u 6m)<br>(2)Blood test indicator<br>a) CA125: E<C [a](f/u 6m)<br>(3)Menstrual volume score(PBAC): E<C [a](f/u 6m)<br>(4)TCM syndrome score: E<C [a](f/u 6m)<br>(5)Uterine volume: E<C [a](f/u 6m)<br>(6)QOL(SF-36): E>C [a](f/u 6m)<br>(7)Pregnancy rate: E>C [a](f/u 6m)<br>(8)Adverse events: E<C [b](f/u 6m)
Peng(2021) [19]	(1)Dysmenorrhea score<br>a) VAS: E>C [b](f/u 3m), E<C [a](f/u 6m, 12m)<br>b) Incidence of mild pain: E<C [b](f/u 3m), E<C [a](f/u 6m, 12m)<br>c) Incidence of severe pain: E<C [b](f/u 3m), E<C [a](f/u 6m, 12m)<br>(2)Blood test indicator<br>a) E2: E<C [b](f/u 3m), E<C [a](f/u 6m, 12m)<br>b) PGF2α: E<C [b](f/u 3m), E<C [a](f/u 6m, 12m)<br>(3)TER: E>C [a](f/u 12m)<br>(4)Menstrual volume score: E<C [b](f/u 3m), E<C [a](f/u 6m, 12m)<br>(5)Uterine volume: E>C [b](f/u 3m), E<C [a](f/u 6m, 12m)<br>(6)Endometrial lesion volume: E<C [b] (f/u 3m), E<C [a] (f/u 6m, 12m)<br>(7)Adverse events: E<C [a](f/u 12m)<br>(8)Recurrence rate: E<C [a](f/u 12m)
Shi(2023) [20]	(1)Dysmenorrhea score(VAS): E<C [a](f/u 3m, 6m)<br>(2)Blood test indicator<br>a) CA125: E<C [a](f/u 3m, 6m)<br>b) Hb: E>C [a](f/u 3m, 6m)<br>(3)TER: E>C [b]<br>(4)Menstrual volume score: E<C [a](f/u 3m, 6m)<br>(5)TCM syndrome score<br>a) Lumbar and knee soreness and weakness: E<C [a]<br>b) Menstrual lumbosacral pain: E<C [a]<br>c) Menstrual abdominal pain: E<C [a]<br>(7)Endometrial lesion volume: E<C [a](f/u 3m, 6m)
Wang(2025) [21]	(1)Operation condition<br>a) Average treatment power: E<C [b]<br>b) Average treatment intensity: E<C [b]

	c) Operation time: E>C [b]<br>(2)Intensity of pain during and post operation(VAS)<br>a) Frequency during operation: E<C [a]<br>b) Highest score during operation: E<C [a]<br>c) 2h post-operation: E<C [a]<br>d) 24h post-operation: E<C [a]<br>(3)Dosage of sedative and analgesic drugs<br>a) Sedative drugs during operation: E<C [a]<br>b) Analgesic drugs during operation: E<C [a]<br>c) Analgesic drugs within 24h post-operation: E<C [a]<br>(4)Postoperative HPOI<br>a) Pain experience: E<C [a]<br>b) Emotion of pain: E<C [a]<br>c) Impact of pain for body and daily life: E<C [a]<br>d) Satisfaction of pain control: E>C [a]<br>e) Satisfaction scores for HPOI pain education: E>C [b]<br>(5)Adverse events(Postoperative complication): E<C [a]
Xu(2019) [22]	(1)Dysmenorrhea score(VAS): E<C [a](f/u 3m), E<C [b](f/u 6m)<br>(2)TER: E>C<br>(3)Menstrual volume score(PBAC): E<C [a](f/u 3m), E<C [b](f/u 6m)<br>(4)Endometrial thickness: E<C [a](f/u 3m), E<C [b](f/u 6m)<br>(5)Adverse events(Postoperative complication): E<C [a]
Xue(2023) [23]	(1)Dysmenorrhea score(VAS): E<C [a]<br>(2)Blood test indicator<br>a) C3: E<C [a]<br>b) C4: E<C [b]<br>c) IgA: E<C [a]<br>d) IgG: E>C [a]<br>e) IgM: E<C [b]<br>f) CA125: E<C [a]<br>(3)Menstrual volume score(PBAC): E<C [a]<br>(4)TCM syndrome score: E<C [a]<br>(5)Uterine volume: E<C [a]<br>(6)Adverse events: E<C [b]
Yi(2024) [24]	(1)Dysmenorrhea score(VAS): E<C [a](f/u 6m)<br>(2)TER: E>C [a]<br>(3)Menstrual volume score(PBAC): E<C [a](f/u 6m)<br>(4)TCM syndrome score: E<C [a](f/u 6m)<br>(5)Uterine volume: E<C [a](f/u 3m, 6m)<br>(6)Endometrial lesion volume: E<C [a](f/u 3m,6m)<br>(7)Cumulative incidence of low estrogen reaction: E<C [a]<br>(8)Adverse events: E<C [a]\
Yu(2017) [25]	(1)Dysmenorrhea score(VAS): E<C [a](f/u 3m)<br>(2)Endometrial lesion volume: E<C [a](f/u 3m)<br>(3)Adverse events: E>C [b]
Zhang(2021) [26]	(1)Blood test indicator(f/u 10d)<br>a) CA125: E<C [a]<br>b) VEGF: E<C [a]<br>c) sVCAM-1: E<C [a]<br>d) IL-1: E<C [a]<br>e) IL-6: E<C [a]<br>f) PGE2: E<C [a]<br>(2)TER: E>C [a](f/u 10d)<br>(3)TCM syndrome score: E<C [a](f/u 10d)
Zhang(2023) [27]	(1)Dysmenorrhea score(VAS): E<C [a]<br>(2)Blood test indicator<br>a) TNFα: E<C [a]<br>b) IL-6: E<C [a]<br>c) E2: E<C [a]<br>d) CA125: E<C [a]<br>(3)TER: E>C [a]<br>(4)TCM syndrome score: E<C [a]<br>(5)Menstrual volume: E<C [a]

	(6)Menstrual cycle: E<C [a]<br>(7)Adverse events: E<C [b]
Zhou(2021) [28]	(1)Blood test indicator(f/u 6m)<br>a) CA125: E<C [a]<br>b) LH: E<C [a]<br>c) FSH: E<C [a]<br>d) PRL: E<C [a]<br>e) E2: E<C [a]<br>(2)TER: E>C [a]<br>(3)Uterine volume: E<C [a](f/u 6m)<br>(4)Endometrial lesion volume: E<C [a](f/u 6m)<br>(5)AFC: E>C [a](f/u 6m)

a = *P*<.05, b = *P*>.05, d = days, h = hours, m = months, AFC = Antral Follicle Count, CMSS = Chinese Medicine Symptoms Score, HPOI = Houston Pain Outcome Instrument, PBAC = Pictorial Blood loss Assessment Chart, QOL = Quality of Life, SF-36 = Short Form-36, TCM syndrome score = Traditional Chinese Medicine syndrome score, TER = Total Effective Rate, VAS = Visual Analogue Scale, C3 = Complement component 3, C4 = Complement component 4, CA125 = Carbohydrate Antigen 125, CRP = C Reactive Protein, E2 = Estradiol, FSH = Follicle Stimulating Hormone, Hb = Hemoglobin, IL-1 = Interleukin-1, IL-6 = Interleukin-6, IgA = Immunoglobulin A, IgM = Immunoglobulin M, IgM = Immunoglobulin G, LH = Luteinizing Hormone, PGE2 = Prostaglandin E2, PGF2α = Prostaglandin F2alpha, PRL = Prolactin, SAA = Serum Amyloid A, sVACM-1 = soluble Vascular cell adhesion protein 1, TNFα = Tumor necrosis factor alpha, VEGF = Vascular Endothelial Growth Factors
