# Supplementary material for: The Efficacy and Safety Herbal Medicine for Symptom Management After HIFU Treatment in Adenomyosis: A Systematic Review and Meta-Analysis
Source: Pharmaceuticals (Basel). 2025 Jun 4;18(6):843. doi: 10.3390/ph18060843 (PMC12195669; doi:10.3390/ph18060843)
Supplement: Supplementary file 1 [file pharmaceuticals-18-00843-s001.zip › Supplementary File S7. Sensitivity analysis .pdf]

**Supplementary File 7A.** Sensitivity analysis for CA125

| Excluding study | SMD | 95%CI | $I^2$ (%) | *p*-value |
|---|---|---|---|---|
| An 2022 | -1.19 | -2.02, -0.35 | 94 | <0.00001 |
| Pang 2022 | -1.69 | -2.69, -0.69 | 96 | <0.00001 |
| Shi 2023 | -1.57 | -2.67, -0.46 | 96 | <0.00001 |
| Xue 2023 | -1.46 | -2.56, -0.37 | 96 | <0.00001 |
| Zhang 2021 | -1.67 | -2.69, -0.66 | 96 | <0.00001 |
| Zhang 2023 | -1.19 | -2.05, -0.34 | 95 | <0.00001 |
| Zhou 2021 | -1.69 | -2.69, -0.69 | 96 | <0.00001 |

SMD=Standardized Mean Difference, CI=confidence interval

**Supplementary File 7B.** Sensitivity analysis for the dysmenorrhea

| Excluding study | MD | 95%CI | $I^2$ (%) | *p*-value |
|---|---|---|---|---|
| An 2022 | -0.42 | -0.96, 0.13 | 96 | <0.00001 |
| Dong 2024 | -0.74 | -1.19, -0.29 | 94 | <0.00001 |
| Pang 2022 | -0.56 | -1.13, 0.02 | 97 | <0.00001 |
| Peng 2021 | -0.55 | -1.26, 0.16 | 96 | <0.00001 |
| Shi 2023 | -0.63 | -1.25, -0.01 | 96 | <0.00001 |
| Xu 2019 | -0.71 | -1.24, -0.17 | 96 | <0.00001 |
| Xue 2023 | -0.55 | -1.12, 0.03 | 97 | <0.00001 |
| Yi 2024 | -0.53 | -1.09, 0.03 | 97 | <0.00001 |
| Yu 2017 | -0.35 | -0.87, 0.18 | 96 | <0.00001 |
| Zhang 2023 | -0.42 | -0.96, 0.13 | 96 | <0.00001 |

MD=Mean Difference, CI=confidence interval
